# Condyloma lata in an HIV patient with secondary syphilis masquerading as anal condyloma—a case report

**DOI:** 10.1007/s00384-024-04678-9

**Published:** 2024-07-22

**Authors:** Abhinandan Anand Raman, Jena Auerbach, Gary P. Wang

**Affiliations:** 1 Division of Infectious Diseases and Global Medicine, Department of Medicine, University of Florida, Gainesville, FL USA; 2 Pathology and Laboratory Medicine Service, North Florida/South Georgia Veterans Health System, Gainesville, FL USA; 3 Medical Service, North Florida/South Georgia Veterans Health System, Gainesville, FL USA

## Case presentation

**Fig. 1:**
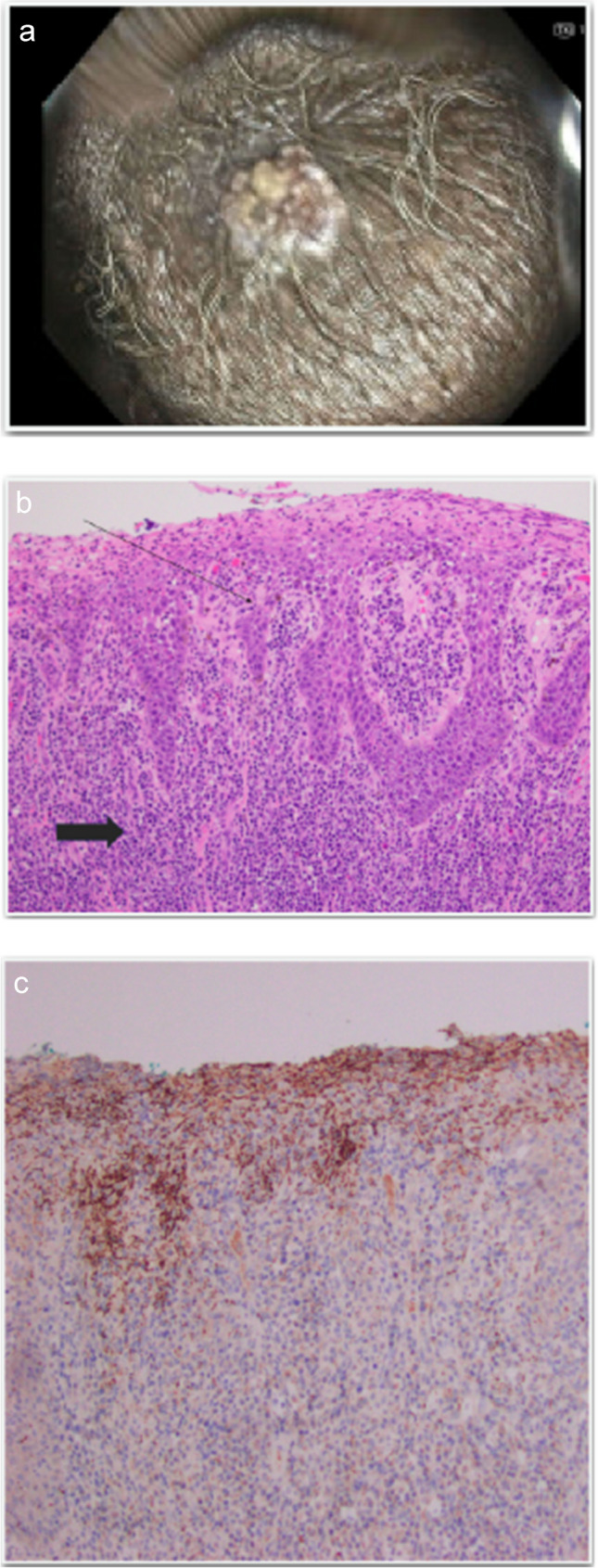
**A** Anal condyloma seen during colonoscopy. **B** H&E stain (100 × magnification). Long arrow highlights elongated rete ridges of an acutely inflamed epidermis. Thick arrow shows the plasma cell infiltrate. **C** Spirochetes within the epidermis are highlighted by a *Treponema pallidum* immunohistochemical stain (brown)

A 67-year-old man with HIV infection well-controlled on once-daily bictegravir-tenofovir alafenamide-emtricitabine presented to primary care with several months of throbbing perianal pain, which he had attributed to hemorrhoids. He denied accompanying symptoms such as unintentional weight loss, changes in stool consistency, constipation, or abdominal discomfort. Following referral from his primary care physician (PCP), he underwent colonoscopy which showed internal hemorrhoids and an external perianal condyloma (Fig. 1A). He was referred to general surgery for excision of the anal condyloma. The surgical biopsy specimen showed ulcerated condyloma acuminatum with lymphoplasmacytic inflammatory infiltrate (Fig. 1B). Due to the presence of plasma cells, immunohistochemical stain was performed which revealed numerous spirochetes at the epithelial and subepithelial junction (Fig. 1C). Infectious diseases service was consulted for further management.

The patient reported a remote history of syphilis for which he was treated with intramuscular penicillin. His laboratory tests from 11 months prior to presentation showed positive syphilis IgG/IgM, positive *Treponema pallidum* particle agglutination (TPPA), and non-reactive rapid plasma reagin (RPR), consistent with past infection. He denied prior history of receptive anal intercourse but disclosed that he had condomless sex with a female sex worker approximately 4 months prior, which coincided with the onset of his perianal pain. Laboratory studies confirmed positive syphilis IgG/IgM and positive TPPA, and an RPR titer of 1:1024. He was treated with a single intramuscular injection of benzathine penicillin 2.4 MU for secondary syphilis.

## Discussion

The presentation of secondary syphilis can be variable and often includes rash, lymphadenopathy, condylomata lata, alopecia, periostitis, and/or hepatitis [1]. However, when patients present with condylomata lata in the absence of other signs and symptoms, syphilis may not be considered in the differential diagnosis initially and the lesion may be misdiagnosed as condylomata acuminata, hemorrhoids, or anal cancer [2].

Tayal et al. [2] reported a case of a 49-year-old male with perianal lesions that were suspicious for malignancy. Anal cancer was excluded, and a diagnosis of syphilis was confirmed with histopathology, imaging, and syphilis serology. Bruins et al. [3] described a 41-year-old man who presented to a dermatology clinic with non-painful anal papules after protected anal contact with multiple male partners. The differential diagnosis included HPV-associated genital warts and syphilis. Biopsy of the lesion showed numerous spirochetes and nucleic acid amplification analysis of the tissue was negative for HPV. Diagnosis of syphilis was later confirmed with serology. He was treated with intramuscular penicillin and the lesion resolved completely at follow-up. Cox et al. [4] reported a case of a 29-year-old woman presenting to a surgical outpatient clinic with symptoms of perianal fissure. The fissures were debrided, and the histological examination was consistent with syphilis. Thayer et al. [4]. Thayer et al. [5] described a 35-year-old woman with a new diagnosis of HIV and exophytic vulvar and perianal lesions with inguinal lymphadenopathy, concerning for vulvar and anal carcinoma. She was diagnosed with presumed late latent syphilis based on histopathologic examination of the biopsy specimen and imaging studies. She was treated with weekly intramuscular penicillin G benzathine and had near complete resolution of her lesions. As in these case reports, the present case highlights the challenges of recognizing condylomata lata in patients without signs and symptoms suggestive of syphilis and underscores the importance of considering syphilis in the differential diagnosis of anal and genital lesions.

## Conclusion

Condylomata lata in the anogenital area may resemble HPV-associated warts, malignancy, or other anogenital lesions. Considering syphilis in the differential diagnosis and promptly obtaining the affected tissues for histopathological examination and serologic testing are critical to confirm the diagnosis.

## Data Availability

No datasets were generated or analyzed during the current study.
